# Association of maternal phthalates exposure and metabolic gene polymorphisms with congenital heart diseases: a multicenter case-control study

**DOI:** 10.1186/s12884-024-06343-z

**Published:** 2024-02-26

**Authors:** Nana Li, Hong Kang, Zhen Liu, Lu Li, Ying Deng, Meixian Wang, Yuting Li, Wenli Xu, Xiaohong Li, Yanping Wang, Jun Zhu, Jing Tao, Ping Yu

**Affiliations:** 1 National Center for Birth Defect Monitoring, West China Second University Hospital, Sichuan University, Sec.3 No.17, South RenMin Road, Chengdu, Sichuan China; 2 Key Laboratory of Birth Defects and Related Diseases of Women and Children (Sichuan University), Ministry of Education, Chengdu, Sichuan China; 3 Department of Maternal Healthcare, Pidu Maternal and Child Care Hospital, Chengdu, China

**Keywords:** Congenital heart diseases, Phthalates, Uridine diphosphate (UDP) glucuronosyl transferase family 1 member A7 (*UGT1A7*), Interaction

## Abstract

**Background:**

The majority of congenital heart diseases (CHDs) are thought to result from the interactions of genetics and the environment factors. This study aimed to assess the association of maternal non-occupational phthalates exposure, metabolic gene polymorphisms and their interactions with risk of CHDs in offspring.

**Methods:**

A multicenter case-control study of 245 mothers with CHDs infants and 268 control mothers of health infant was conducted from six hospitals. Maternal urinary concentrations of eight phthalate metabolites were measured by ultra-high performance liquid chromatography coupled with tandem mass spectrometry (UHPLC-MS/MS). Twenty single nucleotide polymorphisms (SNPs) in cytochrome P450 family 2 subfamily C member 9 (*CYP2C9*) and 19 (*CYP2C19*), uridine diphosphate (UDP) glucuronosyl transferase family 1 member A7 (*UGT1A7*), family 2 member B7 (*UGT2B7*) and B15(*UGT2B15*) genes were genotyped. The multivariate logistic regressions were used to estimate the association between maternal phthalates exposure or gene polymorphisms and risk of CHDs. Generalized multifactor dimensionality reduction (GMDR) was used to analyze the gene–gene and gene–phthalates exposure interactions.

**Results:**

There was no significant difference in phthalate metabolites concentrations between the cases and controls. No significant positive associations were observed between maternal exposure to phthalates and CHDs. The SNPs of *UGT1A7* gene at rs4124874 (under three models, log-additive: aOR = 1.74, 95% CI:1.28–2.37; dominant: aOR = 1.86, 95% CI:1.25–2.78; recessive: aOR = 2.50, 95% CI: 1.26–4.94) and rs887829 (under the recessive model: aOR = 13.66, 95% CI: 1.54–121) were significantly associated with an increased risk of CHDs. Furthermore, the associations between rs4124874 (under log-additive and dominant models) of *UGT1A7* were statistically significant after the false discovery rate correction. No significant gene-gene or gene-phthalate metabolites interactions were observed.

**Conclusions:**

The polymorphisms of maternal *UGT1A7* gene at rs4124874 and rs887829 were significantly associated with an increased risk of CHDs. More large-scale studies or prospective study designs are needed to confirm or refute our findings in the future.

**Supplementary Information:**

The online version contains supplementary material available at 10.1186/s12884-024-06343-z.

## Introduction

Congenital heart diseases (CHDs), typically defined as structural and functional abnormalities of the heart and great vessels during embryonic development, are the most common type of birth defects. The reported birth prevalence of CHDs varies widely across countries and continents. A recent meta-analysis has estimate that the worldwide prevalence of CHDs in live births increased to 9.41/‰ in the period 2010–2017 [1]. In China, total CHDs birth prevalence increased continuously, from 0.201‰ in 1980–1984 to 4.905‰ in 2015–2019 [2]. CHDs are the leading cause of infant morbidity and mortality, and account for approximately 40% of prenatal deaths and 20% of mortality in the first year of life [3]. Therefore, CHDs place a heavy burden on the healthcare system, and have become a sizable public health concern. The etiology of CHDs is complex and multifactorial. To date, $\sim$20–30% of the CHDs cases could be identified with clear environmental or genetic factors, most CHDs cases are considered to be caused by the interaction of genetic and environmental factors [4–6].

Phthalates, are a family of synthetic chemicals widely used as plasticizers in many consumer products. Low-molecular-weight phthalates, such as di-n-butyl phthalate (DBP) and di-isobutyl phthalate (DiBP), are present in fragrances, pharmaceuticals, cosmetics, personal-care products, and packaging materials, whereas high-molecular-weight phthalates, such as di-2-ethylhexyl phthalate (DEHP) and benzyl-n-butyl ortho-phthalate (BBP), are used to soften plastics, particularly PVC building materials [7, 8]. Phthalates are not covalently bound to other substances and can easily permeate and migrate into environmental media, as result, the general population is ubiquitously exposed to phthalates via oral, dermal, inhalation, and intravenous routes [9, 10]. Phthalate metabolites have been consistently detected in urine from the general population including pregnant women worldwide [7–9, 11]. Between 2000 and 2017, indoor phthalate exposure in China has led to 3.32 million disability-adjusted life years (DALYs) per year, accounting for 0.90% of total DALYs across China, with DBP, DiBP and DEHP being the most abundant phthalates in indoor environments of residences, offices, and schools [12].

Phthalates can easily cross the placental barrier and impact the developing fetus in utero [7]. Growing literatures have reported that prenatal exposure to phthalates were associated with the risk of multiple adverse pregnancy outcomes, including preterm birth [13, 14], spontaneous pregnancy loss [15, 16], low birth weight [17], male newborn genital anomalies [18], and fetal retarded growth [19]. Two animal experiment have observed that BBP or DBP exposure could cause abnormalities in zebrafish embryo morphology, including cardiac structure deformities [20, 21]. To date, population epidemiological studies on the association between maternal occupational phthalates exposure and CHDs are limited, and the results are inconsistent. For example, two case-control studies have shown that maternal occupational phthalates exposures are associated with increased risk of total CHDs [22] or some CHDs subtypes [23]. However, three case-control studies have not found significant association between maternal occupational phthalates exposure to and CHDs [24–26]. Besides, phthalates exposure is assessed through subjective reporting, which may result in recall bias and not fully objectively reflect the exact level in vivo. To our knowledge, there have no studies on the association between maternal non-occupational phthalates exposure, identified by the biomarker phthalates, and the risk of CHDs.

The metabolism of environmental exposure is the first step to affect the occurrence of disease, and the individual metabolism level can significantly affect the risk of disease occurrence. The cytochrome P450 enzymes (CYPs), such as *CYP2C9*, and *CYP2C19*, and uridine diphosphate (UDP) -glucuronyl transferases (UGTs), such as *UGT1A7*, *UGT2B7* and *UGT2B15*, have been proposed to play important roles in the phase I and phase II biotransformation of phthalates, respectively [7, 27, 28]. Common single nucleotide polymorphisms (SNPs) genetic polymorphisms in these genes could affect individual susceptibility to adverse effects of exposure to phthalates. One study suggested that the SNPs of UGT, such as rs7439366 of *UGT2B7*, rs1902023 of *UGT2B15*, were associated with the clearance of bisphenol A and phthalates in in patients with polycystic ovary syndrome [29]. One study observed that rs1799853 and rs1057910 of *CYP2C9* could reduce DEHP biotransformation, and rs1799853 and rs1057910 of *CYP2C9*, rs12248560 of *CYP2C19*, and rs11692021of *UGT1A7* might represent biomarkers of susceptibility or resilience in phthalates exposure [28]. However, few studies have investigated maternal genetic susceptibility to CHDs related to phthalates. Additionally, few studies have explored possible gene-environment interactions.

In the present study, we first evaluated the association between maternal exposure to phthalates by measuring urinary levels of phthalate metabolites during the second pregnancy and the risk of fetal CHDs. Then, we investigated the association between maternal genetic polymorphisms and the risk of fetal CHDs. Finally, we explored the potential interaction between maternal genetic variants and exposure to phthalates on the risk of CHDs.

## Methods

### Study population

This study was based on the project of a multicenter hospital-based case-control study which was performed from February 2010 to July 2015. The subjects were recruited from six tertiary maternal and child health hospitals with the qualification of performing prenatal diagnoses, located in Fuzhou, Nanning, Chengdu, Zhengzhou, Wuhan and Shenzhen city, respectively.

The cases group were pregnant women whose fetuses were diagnosed with CHDs by echocardiography and no any extracardiac abnormalities and with gestational age more than 12 weeks. CHDs were further confirmed by humanitarian examination of the pathological anatomy for aborted fetuses or ultrasound examination performed within 30 postnatal days and telephone follow-up performed within 60 days for liveborn fetuses. The exclusion criteria were as follows: (1) fetuses with syndromic diseases and chromosomal aberrations, (2) pregnant women with multiple pregnancies, (3) a mother with a family history of CHDs.

The control group were pregnant women with a singleton pregnancy without major congenital malformations diagnosed by echocardiography in the same hospital and with gestational age more than 12 weeks. A further ultrasound examination was performed within 30 postnatal days, and a telephone follow-up was performed within 60 days.

Each participant participated in face-to-face interview. The structured questionnaire was composed of parental social demographics, living environment and habits, working environment, maternal reproductive history, maternal illness and drug use history, maternal diet and nutrition, and maternal life events and mental state.

Four millilitres of blood was collected in EDTA from each participant during the second trimester by venepuncture and stored at − 70℃until genotyping. Ten millilitres single-spot urine samples of each participant during the second trimester was collected in a nonsterile clean polypropylene container, then divided into aliquots and stored at − 80 °C until further analysis.

### Measurement of urinary concentrations of phthalate metabolites

Phthalate metabolites concentration was measured at the West China School of Public Health, Sichuan University by ultra-high performance liquid chromatography coupled with tandem mass spectrometry (UHPLC-MS/MS). Eight phthalate metabolites from four parent compounds were quantified, including mono-n-butyl phthalate (MnBP, metabolite of di-n-butyl phthalate (DnBP)); mono-isobutyl phthalate (MiBP, metabolite of diisobutyl phthalate (DiBP)); mono-benzyl phthalate (MBzP, metabolite of butylbenzyl phthalate (BBzP)); five metabolites of di (2-ethylhexyl) phthalate (DEHP):[mono(2-ethyl-hexyl) phthalate (MEHP),mono(2-ethyl-5-hydroxyhexyl) phthalate (MEHHP),mono(2-ethyl-5-oxohexyl) phthalate (MEOHP), mono (2-ethyl-5-carboxypentyl)phthalate (MECCP), and mono-2- carboxymethyl hexyl phthalate (MCMHP)].

The analysis was performed using an ultra-high performance liquid chromatography ACQUITY UPLC I-Class coupled to an Xevo TQ-XS triple stage quadrupole mass spectrometer (Waters, USA). The details regarding the preparation and analysis of the samples are available in Supplementary Appendix A.

To adjust the dilution of urine, creatinine-adjusted concentrations of urinary phthalate metabolites were calculated. The concentrations below the limit of detection (LOD) were replaced by LOD/$\sqrt{2}$.

### DNA extraction and genotyping

Genomic DNA was extracted with magnetic bead method (BioTeke, Wuxi, China) according to the recommended protocol. Twenty SNPs in the *UGT1A7*, *UGT2B7*, *UGT2B15*, *CYP2C19*, *CYP2C9* were selected based on the following principal criteria: (1) an association with diseases in previous studies or the metabolic level of phthalates [28–44], (2) a minor allele frequency > 0.05 in Han Chinese. In total, 20 SNPs were selected. The genotypes of these SNPs were detected using multiple-polymerase chain reaction amplification and next generation sequencing (iGeneTech Bioscience Co., Ltd, Beijing, China). More detailed information about the studied genetic variants and genotyping is presented in Supplementary Appendix, Table S2.

For quality-control assessment, genotyping was repeated in 10% of samples, and the consistency rate was 100%.

### Statistical analyses

The composition ratio of baseline characteristics between case and control groups was compared by χ^2^ test using Statistical Package for Social Sciences (SPSS) version 16.0 software (SPSS Inc., IBM, Chicago, USA).These characteristics included maternal age (at the time of the last menstrual period), maternal ethnicity, maternal education level, parental smoking or environmental tobacco smoke (ETS) exposure, maternal alcohol consumption, parity, maternal pre-pregnancy body mass index (ppBMI) (BMI = weight/(height × height) (kg/m^2^) before known pregnancy), maternal folic acid supplement.

As the distributions of phthalate metabolites concentrations did not meet the normality assumption, they were described as median (interquartile range) and compared with Mann–Whitney U test. The concentrations of phthalate metabolites were performed natural log_10_ transformation and further divided into three categories according to the tertile which they fell into. Logistic regression analysis was used to estimate the associations of the levels of phthalate metabolites with CHDs using SPSS version 16.0 software (SPSS Inc., IBM, Chicago, USA).

Hardy–Weinberg equilibrium was assessed in the controls using Plink software (http://zzz.bwh.harvard.edu/plink/ld.shtml). Logistic regression analysis was performed to investigate the association between individual genetic polymorphisms and CHDs using Plink software.

The effects of the gene–gene and gene-phthalates exposure interactions on CHDs occurrence were evaluated by logistic models using generalized multifactor dimensionality reduction (GMDR, version 0.7, University of Virginia, Charlottesville, VA).

All analyses were adjusted for covariates or potential confounders. False discovery rate (FDR) correction of multiple hypothesis testing was performed. Two-sided *P* < 0.05 was considered statistically significant.

## Results

### Characteristics of the study participants

In this study, a total of 513 subjects were analyzed, including 245 cases with CHDs fetuses and 268 controls. The baseline characteristics of participants were listed in Table 1.

**Table 1:** Descriptive characteristics of the participants

Variable/Characteristic	Controls (*n* = 268)	CHDs cases (*n* = 245)	χ^2^	P-values
	No. (%)	No. (%)		
Maternal age (years)			1.067	0.302
< 35	234(87.31%)	221(90.2%)		
≥ 35	34(12.69%)	24(9.8%)		
Maternal ethnicity			43.979	< 0.001
Han	259(96.64%)	189(77.14%)		
Others	9(3.36%)	56(22.86%)		
Maternal education level			63.325	< 0.001
Junior school or lower	37(13.81%)	101(41.22%)		
High school	42(15.67%)	52(21.22%)		
College or higher	189(70.52%)	92(37.55%)		
Parental smoking or ETS exposure^a^			6.524	0.011
Yes	124(46.27%)	141(57.55%)		
No	144(53.73%)	104(42.45%)		
Maternal alcohol consumption^a^			0.321	0.571
Yes	41(15.3%)	42(17.14%)		
No	227(84.7%)	203(82.86%)		
Parity			15.727	< 0.001
Nullipara	143(53.36%)	88(35.92%)		
Multipara	125(46.64%)	157(64.08%)		
Pre-pregnancy BMI (kg/m^2^)			0.283	0.868
≤ 18.5	60(22.39%)	56(22.86%)		
18.5–24	183(68.28%)	163(66.53%)		
≥ 24	25(9.33%)	26(10.61%)		
Folic acid supplements^a^			1.360	0.244
Yes	226(84.33%)	197(80.41%)		
No	42(15.67%)	48(19.59%)		

^a^ The exposure was defined from the 3 months before pregnancy to the second trimester

There were significant differences in the distributions of maternal ethnicity (96.64% Han in controls and 77.14% Han in cases, *P* < 0.001), maternal education level (70.52% controls vs. 37.55% cases had College or higher education, *P* < 0.001), parental smoking or ETS exposure (53.73% controls vs. 42.45% cases, *P* = 0.011).

Compared with control mothers, CHDs case mothers were also more likely to be multipara (46.64 controls vs.64.08% cases, *P* < 0.001). Maternal age, maternal alcohol consumption, pre-pregnancy BMI and folic acid supplements did not show statistical differences between the two groups (*P*_all_>0.05).

### Distribution of phthalate metabolite concentrations in maternal urine

The LODs, detection rates, and distributions of phthalate metabolites were summarized in Table 2. The LOD for MiBP, MnBP, MBzP, MEHP, MEHHP, MEOHP, MECPP and MCMHP was 0.50 ng/mL, 0.50 ng/mL, 0.05 ng/mL, 0.50 ng/mL, 0.05 ng/mL, 0.05 ng/mL, 0.10 ng/mL and 0.20 ng/mL, respectively. In addition to relatively low detection rate of 84.60% for MBzP, the detection rates of other seven metabolites were nearly or equal to 100%.

**Table 2:** Urinary concentrations of unadjusted (ng/mL) and adjusted PAEs (µg/g Cr)

Diether phthalate	Phthalate metabolites (µg/g creatinine)	LOD (ng/mL)	Concentration≥ LOD, n (%)	Median (IQR)			P-Value^a^
				Total participants (*n* = 529)	Controls (*n* = 268)	CHDs Cases (*n* = 245)	
DiBP	MiBP	0.50	511(99.61%)	10.23(5.94,17.43)	10.43(6.09,18.77)	10.01(5.53,16.12)	0.452
DnBP	MnBP	0.50	513(100%)	36.9(17.58,71.91)	34.08(16.6,68.19)	41.11(19.02,76.23)	0.106
BBzP	MBzP	0.05	434(84.60%)	0.08(0.04,0.23)	0.09(0.04,0.19)	0.08(0.04,0.28)	0.407
DEHP	MEHP	0.50	492(95.90%)	2.53(1.30,4.58)	2.71(1.41,4.95)	2.37(1.19,4.04)	0.052
	MEHHP	0.05	513(100%)	2.45(1.39,4.11)	2.35(1.38,3.85)	2.51(1.41,4.84)	0.137
	MEOHP	0.05	513(100%)	1.44(0.86,2.45)	1.40(0.83,2.3)	1.48(0.88,2.81)	0.176
	MECPP	0.10	513(100%)	5.48(3.29,9.10)	5.43(3.25,8.25)	5.49(3.38,10.36)	0.219
	MCMHP	0.20	513(100%)	1.59(1.12,2.58)	1.60(1.16,2.31)	1.55(1.10,2.89)	0.489

^a^*P* values for the Mann‒Whitney U test between case and control group

The median concentrations of MnBP, MEHHP, MEOHP and MECPP were higher in CHDs than in controls (41.11 vs. 34.08, 2.51 vs. 2.35, 1.48 vs. 1.40, 5.49 vs. 5.43 µg/g creatinine, respectively), whereas the median concentrations of MiBP, MBzP, MEHP and MCMHP were lower in CHDs than in controls (10.01 vs. 10.43, 0.08 vs. 0.09, 2.37 vs. 2.71, 1.55 vs. 1.60 µg/g creatinine, respectively). However, these eight phthalate metabolites concentrations did not show significant differences between two groups (*P*_all_>0.05).

### Associations between maternal phthalates exposure and CHDs risk

The relation between maternal phthalates exposure and the risk of CHDs was displayed in Table 3. Among the eight metabolites, including MiBP, MnBP, MBzP, MEHP, MEHHP, MEOHP, MECPP, MCMHP, the first-tertile log10-transformed concentration of each was used as a reference, the second- and third-tertile concentrations were not associated with the risks of CHDs. Stratified analysis in the Han Chinese maternal population also did not observe significant positive associations between maternal exposure to phthalates and CHDs (Supplementary Appendix Table S3).

**Table 3:** Logistic regression analyses of the association between phthalate metabolites in maternal urinary samples and the risk of CHDs

Elements	Concentration levels ^a^	Controls	CHDs Cases	cOR (95% CI)	aOR (95% CI)^b^
		No. (%)	No. (%)		
MiBP	First-tertile	90(33.58%)	82(33.47%)	Reference	Reference
	Second-tertile	87(32.46%)	85(34.69%)	1.07(0.70–1.64)	1.04(0.65–1.67)
	Third-tertile	91(33.96%)	78(31.84%)	0.94(0.62–1.44)	0.87(0.54–1.41)
MnBP	First-tertile	96(35.82%)	76(31.02%)	Reference	Reference
	Second-tertile	94(35.07%)	76(31.02%)	1.02(0.67–1.57)	1.14(0.69–1.79)
	Third-tertile	78(29.10%)	93(37.96%)	1.51(0.98–2.31)	1.48(0.92–2.38)
MBzP	First-tertile	81(30.22%)	85(34.69%)	Reference	Reference
	Second-tertile	98(36.57%)	78(31.84%)	0.76(0.50–1.16)	0.75(0.46–1.21)
	Third-tertile	89(33.21%)	82(33.47%)	0.88(0.57–1.35)	0.93(0.58–1.51)
MEHP	First-tertile	81(30.22%)	90(36.73%)	Reference	Reference
	Second-tertile	88(32.84%)	83(33.88%)	0.85(0.56–1.30)	0.67(0.41–1.08)
	Third-tertile	99(36.94%)	72(29.39%)	0.66(0.43-1.00)	0.63(0.39–1.02)
MEHHP	First-tertile	92(34.33%)	79(32.24%)	Reference	Reference
	Second-tertile	94(35.07%)	75(30.61%)	0.93(0.61–1.42)	0.76(0.47–1.23)
	Third-tertile	82(30.60%)	91(37.14%)	1.29(0.85–1.97)	1.25(0.77–2.01)
MEOHP	First-tertile	93(34.7%)	77(31.43%)	Reference	Reference
	Second-tertile	90(33.58%)	85(34.69%)	1.14(0.75–1.74)	0.90(0.56–1.45)
	Third-tertile	85(31.72%)	83(33.88%)	1.18(0.77–1.81)	1.09(0.68–1.77)
MECPP	First-tertile	94(35.07%)	76(31.02%)	Reference	Reference
	Second-tertile	86(32.09%)	85(34.69%)	1.22(0.80–1.87)	0.98(0.61–1.59)
	Third-tertile	88(32.84%)	84(34.29%)	1.18(0.77–1.81)	1.01(0.62–1.64)
MCMHP	First-tertile	85(31.72%)	82(33.47%)	Reference	Reference
	Second-tertile	100(37.31%)	74(30.2%)	0.77(0.50–1.18)	0.70(0.43–1.15)
	Third-tertile	83(30.97%)	89(36.33%)	1.11(0.73–1.70)	1.12(0.69–1.81)

^a^Data were divided by overall maternal urine tertiles log10-transformed phthalate metabolites concentrations

^b^aOR, adjusted odds ratio. Logistic regression was used to calculate odds ratios and 95% CIs; all models were adjusted for maternal age (continuous), maternal ethnicity, maternal education level, parental smoking or ETS exposure, maternal alcohol consumption, gravidity, pre-pregnancy BMI (continuous), folic acid supplements

### Association between maternal gene polymorphisms and CHDs risk

The genotype distributions for polymorphisms of *UGT1A7*, *UGT2B15*, *UGT2B7*, *CYP2C19* and *CYP2C9* in the controls were consistent with Hardy-Weinberg equilibrium (see Supplementary Appendix, Table S4).

The association between single gene loci polymorphisms and the risk of CHDs when assuming various genetic models was shown in Table 4. In the *UGT1A7* gene, the SNP rs4124874 was associated with an increased risk of CHDs (under the log-additive model: aOR = 1.74, 95% CI:1.28–2.37; under the dominant model: aOR = 1.86, 95% CI:1.25–2.78; under the recessive model: aOR = 2.50, 95% CI: 1.26–4.94), and the SNP rs887829 was associated with an increased risk of CHDs under the recessive model (aOR = 13.66, 95% CI: 1.54–121). However, for the recessive model, the associations were not statistically significant after the false discovery rate (FDR) correction. No significant association was found between any of the remaining 18 selected loci and the risk of CHDs.

**Table 4:** Association between maternal genotypes and the risk of CHDs

Gene	dbSNP_ID	Model	Genotype	Controls	Cases	aOR^a^	P-Value	FDR-BH P-Value
				No. (%)	No. (%)			
*UGT1A7*	rs11692021	Log-additive	-	-	-	0.79(0.56–1.11)	0.1754	0.7015
		Dominant	T/T	154(57.46)	159(64.9)	1		
			T/C-C/C	114(42.54)	86(35.1)	0.75(0.49–1.13)	0.1632	0.6298
		Recessive	T/T-T/C	252(94.03)	234(95.51)	1		
			C/C	16(5.97)	11(4.49)	0.78(0.31–1.93)	0.5863	0.7818
*UGT1A7*	rs4124874	Log-additive	-	-	-	**1.74(1.28–2.37)**	0.0004	0.009*
		Dominant	T/T	142(52.99)	96(39.18)	1		
			T/G-G/G	126(47.01)	149(60.82)	**1.86(1.25–2.78)**	0.0023	0.0469*
		Recessive	T/T-T/G	250(93.28)	208(84.9)	1		
			G/G	18(6.72)	37(15.1)	**2.50(1.26–4.94)**	0.0085	0.1253
*UGT1A7*	rs10929302	Log-additive	-	-	-	1.23(0.80–1.90)	0.3456	0.7325
		Dominant	G/G	215(80.22)	194(79.18)	1		
			G/A-A/A	53(19.78)	51(20.82)	1.04(0.64–1.70)	0.8754	0.9423
		Recessive	G/G-G/A	267(99.63)	236(96.33)	1		
			A/A	1(0.37)	9(3.67)	13.66(1.54–121)	0.0188	0.1253
*UGT1A7*	rs887829	Log-additive	-	-	-	1.22 (0.79–1.88)	0.3663	0.7325
		Dominant	C/C	214(79.85)	194(79.18)	1		
			C/T-T/T	54(20.15)	51(20.82)	1.03(0.63–1.68)	0.9094	0.9423
		Recessive	C/C-C/T	267(99.63)	236(96.33)	1		
			T/T	1(0.37)	9(3.67)	**13.66(1.54–121)**	0.0188	0.1253
*UGT1A7*	rs4148323	Log-additive	-	-	-	0.69 (0.47–1.01)	0.0577	0.5055
		Dominant	G/G	174(64.93)	181(73.88)	1		
			G/A-A/A	94(35.07)	64(26.12)	0.70(0.45–1.08)	0.1095	0.6298
		Recessive	G/G-G/A	255(95.15)	240(97.96)	1		
			A/A	13(4.85)	5(2.04)	0.38 (0.11–1.29)	0.1199	0.5994
*UGT2B15*	rs3100	Log-additive	-	-	-	1.38 (0.95–2.01)	0.0888	0.5055
		Dominant	G/G	193(72.01)	160(65.31)	1		
			G/A-A/A	75(27.99)	85(34.69)	1.41 (0.92–2.16)	0.1113	0.6298
		Recessive	G/G-G/A	262(97.76)	238(97.14)	1		
			A/A	6(2.24)	7(2.86)	1.83(0.55–6.10)	0.3281	0.7149
*UGT2B15*	rs4148269	Log-additive	-	-	-	1.37(0.94–1.99)	0.1011	0.5055
		Dominant	T/T	192(71.64)	160(65.31)	1		
			T/G-G/G	76(28.36)	85(34.69)	1.39(0.91–2.13)	0.1284	0.6298
		Recessive	T/T-T/G	262(97.76)	238(97.14)	1		
			G/G	6(2.24)	7(2.86)	1.83(0.55–6.10)	0.3281	0.7149
*UGT2B15*	rs2045100	Log-additive	-	-	-	0.93(0.68–1.26)	0.6269	0.7973
		Dominant	T/T	125(46.64)	119(48.57)	1		
			T/A-A/A	143(53.36)	126(51.43)	0.89(0.60–1.33)	0.5612	0.9423
		Recessive	T/T-T/A	244(91.04)	223(91.02)	1		
			A/A	24(8.96)	22(8.98)	0.97(0.50–1.88)	0.9232	1
*UGT2B15*	rs1902023	Log-additive	-	-	-	0.93 (0.71–1.23)	0.6104	0.7973
		Dominant	C/C	84(31.34)	85(34.69)	1		
			C/A-A/A	184(68.66)	160(65.31)	0.97(0.64–1.48)	0.8849	0.9423
		Recessive	C/C-C/A	208(77.61)	200(81.63)	1		
			A/A	60(22.39)	45(18.37)	0.83(0.51–1.36)	0.4569	0.7149
*UGT2B15*	rs9994887	Log-additive	-	-	-	0.96(0.73–1.26)	0.7578	0.7973
		Dominant	G/G	85(31.72)	83(33.88)	1		
			G/A-A/A	183(68.28)	162(66.12)	1.02 (0.67–1.56)	0.916	0.9423
		Recessive	G/G-G/A	208(77.61)	199(81.22)	1		
			A/A	60(22.39)	46(18.78)	0.84(0.52–1.38)	0.5004	0.7149
*UGT2B15*	rs13112099	Log-additive	-	-	-	0.96(0.73–1.26)	0.7578	0.7973
		Dominant	G/G	85(31.72)	83(33.88)	1		
			G/T-T/T	183(68.28)	162(66.12)	1.02(0.67–1.56)	0.916	0.9423
		Recessive	G/G-G/T	208(77.61)	199(81.22)	1		
			T/T	60(22.39)	46(18.78)	0.84(0.52–1.38)	0.5004	0.7149
*UGT2B15*	rs7686914	Log-additive	-	-	-	1.04(0.79–1.37)	0.7578	0.7973
		Dominant	T/T	60(22.39)	46(18.78)	1		
			T/C-C/C	208(77.61)	199(81.22)	1.19(0.72–1.94)	0.916	0.9423
		Recessive	T/T-T/C	183(68.28)	162(66.12)	1		
			C/C	85(31.72)	83(33.88)	0.98(0.64–1.49)	0.5004	0.7149
*UGT2B15*	rs7696472	Log-additive	-	-	-	1.05(0.80–1.38)	0.7415	0.7973
		Dominant	G/G	60(22.39)	46(18.78)	1		
			G/A-A/A	208(77.61)	199(81.22)	1.19(0.72–1.94)	0.9423	0.9423
		Recessive	G/G-G/A	183(68.28)	161(65.71)	1		
			A/A	85(31.72)	84(34.29)	0.98(0.65–1.50)	0.5004	0.7149
*UGT2B7*	rs4587017	Log-additive	-	-	-	0.83(0.61–1.13)	0.228	0.7325
		Dominant	T/T	19(7.09)	22(8.98)	1		
			T/G-G/G	249(92.91)	223(91.02)	0.71(0.35–1.45)	0.2988	0.8538
		Recessive	T/T-T/G	121(45.15)	115(46.94)	1		
			G/G	147(54.85)	130(53.06)	0.81(0.54–1.21)	0.3473	0.7149
*UGT2B7*	rs7662029	Log-additive	-	-	-	0.85(0.62–1.16)	0.2996	0.7325
		Dominant	A/A	19(7.09)	22(8.98)	1		
			A/G-G/G	249(92.91)	223(91.02)	0.71(0.35–1.45)	0.4125	0.9423
		Recessive	A/A-A/G	125(46.64)	116(47.35)	1		
			G/G	143(53.36)	129(52.65)	0.85(0.57–1.26)	0.3473	0.7149
*UGT2B7*	rs12233719	Log-additive	-	-	-	0.94(0.65–1.36)	0.7391	0.7973
		Dominant	G/G	184(68.66)	177(72.24)	1		
			G/T-T/T	84(31.34)	68(27.76)	0.95 (0.62–1.46)	0.8184	0.9423
		Recessive	G/G-G/T	259(96.64)	238(97.14)	1		
			T/T	9(3.36)	7(2.86)	0.79(0.25–2.45)	0.6793	0.8491
*UGT2B7*	rs10028494	Log-additive	-	-	-	1.14(0.80–1.62)	0.4861	0.7973
		Dominant	A/A	166(61.94)	152(62.04)	1		
			A/C-C/C	102(38.06)	93(37.96)	1.10(0.73–1.66)	0.658	0.9423
		Recessive	A/A-A/C	260(97.01)	236(96.33)	1		
			C/C	8(2.99)	9(3.67)	1.69(0.58–4.94)	0.3395	0.7149
*CYP2C19*	rs12248560	Log-additive	-	-	-	0.82(0.22–3.12)	0.7719	0.7973
		Dominant	C/C	262(97.76)	239(97.55)	1		
			C/T-T/T	6(2.24)	6(2.45)	0.82(0.22–3.12)	0.7719	0.9423
		Recessive	C/C-C/T	268(100)	245(100)	1		
			T/T	0(0)	0(0)	NA	NA	NA
*CYP2C19*	rs4244285	Log-additive	-	-	-	0.96(0.72–1.29)	0.7973	0.7973
		Dominant	G/G	118(44.03)	108(44.08)	1		
			G/A-A/A	150(55.97)	137(55.92)	0.92(0.62–1.37)	0.6817	0.9423
		Recessive	G/G-G/A	239(89.18)	213(86.94)	1		
			A/A	29(10.82)	32(13.06)	1.03(0.56–1.90)	0.9237	1
*CYP2C9*	rs1057910	Log-additive	-	-	-	1.58(0.72–3.47)	0.2585	0.7325
		Dominant	A/A	256(95.52)	225(91.84)	1		
			A/C-C/C	12(4.48)	20(8.16)	1.76(0.76–4.08)	0.189	0.6298
		Recessive	A/A-A/C	267(99.63)	245(100)	1		
			C/C	1(0.37)	0(0)	0(0-inf)	0.9993	1

^a^aOR, adjusted odds ratio. Logistic regression was used to calculate odds ratios and 95% CIs; all models were adjusted for maternal age (continuous), maternal ethnicity, maternal education level, parental smoking or ETS exposure, maternal alcohol consumption, gravidity, pre-pregnancy BMI (continuous), folic acid supplements

### GMDR analyses for gene–gene and gene–environment interactions on CHDs

The gene-gene and gene-environment interaction model by GMDR was presented in Table 5. The *P* value was determined using the permutation test with 1000 replications. For gene-gene interaction, rs4124874 was a susceptibility locus for CHDs risk. Two-locus to five-locus interaction models were observed, but there were no statistical differences. In addition, for gene-phthalate metabolites interaction, five interaction combinations with no statistical significance were observed.

**Table 5:** GMDR analysis for gene–gene and gene–phthalates exposure interaction models in CHDs

Model	Training Bal. Acc	Testing Bal. Acc	Sign Test (P-Value)	CV Consistency
**Gene-gene interaction**				
rs4124874	0.5690	0.5691	10(0.0010)	10/10
rs1057910 rs4124874	0.5933	0.5397	8(0.0547)	5/10
rs4124874 rs3100 rs2045100 rs12233719	0.6565	0.5292	7(0.1719)	5/10
rs4244285 rs11692021 rs4124874 rs1902023 rs4587017	0.7091	0.4999	4(0.8281)	3/10
**Gene-PAEs phthalates interaction**				
rs11692021 rs1902023 MEHP	0.6161	0.5186	7(0.1719)	3/10
rs4244285 rs11692021 rs4124874 rs1902023 rs12233719 MEHP	0.7740	0.4988	5(0.6230)	2/10
rs4244285 rs11692021 rs4124874 rs9994887 rs7662029 MiBP MEHP	0.8376	0.4832	5(0.6230)	3/10
rs4244285 rs11692021 rs4124874 rs9994887 rs7662029 rs12233719 MnBP MBzP	0.8912	0.5023	4(0.8281)	5/10
rs4244285 rs11692021 rs4124874 rs2045100 rs9994887 rs7662029 MiBP MEHP MCMHP	0.9361	0.4287	1(0.9990)	2/10

*Notes* Training Bal. Acc: training balanced accuracy; Testing Bal. Acc: testing balanced accuracy; CV Consistency: cross validation consistency

## Discussion

In this case-control study, we observed no difference in the concentration of maternal phthalate metabolites between the case and control groups, and did not find significant association between the concentrations of maternal phthalate metabolites and the risk of CHDs, while observed that the SNPs rs4124874 and rs887829 of *UGT1A7* gene were associated with an increased risk of CHDs, but did not find significant gene-gene or gene-phthalate metabolite interactions on CHDs.

Our findings suggested that maternal phthalates exposure was not associated with the risk of CHDs, which was similar to the results of two Netherlands studies showing no association of maternal exposure to phthalates with risk of CHDs [24, 26]. Another case-control study in Hungary also reported that maternal job-exposure matrix (JEM)-assessed and self-reported exposures to phthalates were not associated with the risk of CHDs or subtypes [25]. Moreover, our analysis in the Han Chinese maternal population found no significant positive associations between phthalates exposure to and CHDs. The results of stratified analysis were inconsistent with one previous studies reported in China, which found that maternal occupational exposure to phthalates was associated with a higher incidence of total congenital heart defects, with aOR 1.6 (95% CI: 1.0-2.6) [22]. Similarly, a later Chinese study confirmed that maternal occupation exposure to phthalates was associated with CHDs subtypes, including ventricular septal defect (VSD), atrial septal defect (ASD), patent ductus arteriosus (PDA), pulmonary valve stenosis (PVS) [23]. However, due to the small sample size, we did not perform the association analysis between phthalates exposure with CHDs subtypes. In addition to CHDs, maternal occupational exposure to phthalate obtained by a job exposure matrix was associated with increased risks of hypospadias (OR = 3.12; 95% CI, 1.04–11.46) [45]. First trimester urinary DEHP metabolite concentrations were associated with increased odds of neonatal genital anomaly (OR = 2.54, 95% CI, 1.09–5.92) [18]. One study found that the detection ratio of positive BBP and its metabolites in maternal urine was obviously higher in neural tube defects population than that in normal controls [46]. Overall, there are only a few limited reports on phthalate exposure during pregnancy and birth defects.

It is important to note that studies on the association between maternal phthalates exposure and the risk of CHDs in the Chinese population have shown inconsistent results, which may be due to differences in the way exposure was evaluated. Phthalates exposure is ubiquitous. Two previous China studies focused occupational exposures, which only considered a single source of exposure at work, ignoring comprehensive exposure in the living environment. Moreover, it was difficult to estimate accurately the amount of exposure, the time and the frequency due to recall bias. In addition, the discrepancies across studies may be due to the heterogeneity of populations. For example, two European studies found that maternal occupational phthalates exposure was not associated with CHDs, however, the inverse associations were observed between maternal occupational phthalates exposure and the risk of CHDs in China studies. The conflicting results can likely be explained by differences in sensitivity to the biochemical and toxic effects of phthalates due to genetic polymorphisms.

Accumulating evidence have suggested that inter-individual differences in the ability of the xenobiotic metabolism due to high variability in certain metabolic enzyme activities can influence the effects of environmental exposure on birth defects (e.g., oral clefts, neural tube defects, CHDs) [47–50]. The uridine diphosphate (UDP)-glucuronosyl transferase (UGTs), UGT1A7, UGT2B7 and UGT2B15, catalyze the glucuronidation of multiple substrates. As for the *UGT1A7* gene, rs11692021 polymorphism was related to higher risk of chronic pancreatitis (aOR = 1.76, 95% CI: 1.26–2.46) [30]. In addition, rs4124874 and rs4148323 polymorphism were associated with in increased risk of hyperbilirubinemia [31, 32]. However, rs887829 genotype was related to a decreased risk of hyperbilirubinemia (aOR = 0.55, 95% CI: 0.34–0.89) [32]. As for the *UGT2B7* gene, rs7662029 polymorphism was associated with the withdrawal symptoms in methadone maintenance patients [33]. SNP rs4587017 polymorphism might influence the analgesic effects of fentanyl in the cold pressor-induced pain test [34]. SNP rs12233719 polymorphism was associated with never-smoking female lung cancer risk [35]. As for the *UGT2B15* gene, rs2045100 locus, six SNPs (rs3100, rs4148269, rs9994887, rs13112099, rs7686914 and rs7696472), and rs1902023 polymorphism, showed significant associations with increased risk for prostate cancer [36–38]. In the present study, among 17 SNPs in *UGT1A7*, *UGT2B7* and *UGT2B15*, the polymorphisms of maternal *UGT1A7* gene at rs4124874 (under additive, dominant and recessive models), and rs887829 (under recessive model) were associated with increased CHDs risk. The molecular mechanism remains unclear. It is possible that these two intronic loci affect the alternative spicing of the gene products, or they might be in linkage disequilibrium with other causal loci or genes, thereby affecting the metabolism of phthalates.

Two members of the CYP family, CYP2C9 and CYP2C19 are the main Phase I metabolizing enzymes mediating the toxicity of phthalates, their polymorphisms are associated with the risk of many types of diseases. It has been reported that rs1057910 genotype of *CYP2C9* was related to increased risk of adenoma recurrence (aRR = 1.47, 95% CI: 1.19–1.83) [39], or developing sporadic colorectal carcinoma (aOR = 2.77, 95% CI: 1.1653–4.643) [40]. In addition, for the two common polymorphic loci rs12248560 and rs4244285 of *CYP2C19*, the genotype of rs12248560 was associated with decreased breast cancer risk (aOR = 0.77, 95% CI: 0.65–0.93) [41], while rs4244285 polymorphism was related to higher risk of epilepsy (aOR = 4.24, 95% CI: 2.52–7.15) [42], long-term ischemic stroke events (hazard ratio: 1.64, 95% CI: 1.06–2.53) [43], hypertension (aOR = 2.433, 95% CI: 1.797–3.293) [44]. Meanwhile, several studies have observed that *CYP2C9* rs1057910 polymorphism was associated with increased risk for adenoma recurrence (aRR = 1.47, 95% CI 1.19–1.83) [39], or developing sporadic colorectal carcinoma (aOR = 2.589, 95% CI: 1.549–4.330) [40]. In our study, no significant associations between CHDs risk and genotype were seen for the rs12248560 and rs4244285 of *CYP2C19*, or rs1057910 of *CYP2C9* polymorphisms.

It is now widely believed that most structural birth defects including CHDs are caused by a complex combination of genetic and environmental factors that interact to interfere with morphogenetic processes. More and more studies have reported significant interaction effects between gene-environment interactions for the development of CHDs. One study found maternal dietary factors and cystathionine beta synthase (*CBS*) gene variants (rs2851391, rs234714) interactions were significantly associated with risk of CHDs [51]. One study observed the interaction between maternal tobacco exposure and polymorphisms of the *MTHFD1* gene including rs1950902, rs2236222, rs1256142, rs11849530 and rs2236225, was significantly associated with the risk of CHDs in offspring [52]. One study reported a significantly positive interaction between maternal folic acid supplementation and genetic variation at rs828858 of *MTHFD2* for the risk of CHDs [53]. In our previous study, we found that polymorphisms of maternal GST genes (*GSTM1*, *GSTT1*, *GSTP1*) might modify the association of maternal smoke exposure with CHDs [54]. In addition, we also observed that the polymorphisms of maternal *AHR* rs2158041 and rs7811989, or *UGT1A1* rs4148323 might modify the association of PAHs exposure with CHDs, *CYP1A2* rs4646425 or *CYP2E1* rs915908 polymorphisms significantly interacted with PAHs exposure on CHDs [55, 56]. In the present study, no significantly positive interaction of gene-gene or gene-phthalate metabolites for the risk of CHDs was observed, more large-scale studies or prospective study designs are needed to explore the interactions in the future.

This study has several strengths. First, to the best of our knowledge, this is the first study to evaluate the effect of the interaction between maternal phthalates exposure and maternal gene polymorphism on the risk of CHDs. Second, compared with previous phthalates exposure assessment that relied solely on expert industrial hygienist consensus or the self-reported questionnaire, we used a urinary bio-markers based approach to evaluate the association between maternal phthalates exposure and the risk of CHDs, offering an objective measure of exposure. Finally, the subjects of our study were non-occupational, low-dose phthalates exposed pregnant women, thus, the results can be generalized to all women because the environmental factors can be assessed at the individual level.

However, our study still had several limitations. First, small sample size limited the statistical power; besides, due to a small number of cases, our study could not perform the analysis for type-specific CHDs; future studies with larger sample sizes are warranted to confirm or refute our findings. Second, we only measured phthalate metabolites from a single spot urine sample taken at the second trimester which could not precisely estimate the mother’s long-term exposure level. Thus, future studies are needed to collect multiple urine samples. Third, only maternal phthalates exposure and genetic susceptibilities were considered; future studies are needed to investigate the effects of fetal exposure, fetal genotypes, and the interaction between them on the risk of CHDs.

In conclusion, our analysis results indicated that maternal phthalates exposure was not associated with the risk of CHDs. The polymorphisms of maternal *UGT1A7* gene at rs4124874 and rs887829 were significantly associated with an increased risk of CHDs. No significant gene-gene or gene-phthalate metabolites interactions on CHDs was observed. However, due to the complex pathogenesis of CHDs and the limitation of small sample size, more large-scale studies or prospective study designs are needed to confirm or refute our findings in the future.

### Electronic supplementary material

Below is the link to the electronic supplementary material.

Supplementary Material 1

## Data Availability

The variant data for this study have been deposited in the European Variation Archive (EVA) at EMBL-EBI under accession number PRJEB70402.

## Abbreviations

CHDs: congenital heart diseases
aOR: adjusted odds ratio
95% CI: 95% confidence interval
UHPLC-MS/MS: ultra-high performance liquid chromatography coupled with tandem mass spectrometry
SNPs: single nucleotide polymorphisms
CYP2C9: cytochrome P450 family 2 subfamily C member 9
CYP2C9: cytochrome P450 family 2 subfamily C member 19
UGT1A7: uridine diphosphate (UDP) glucuronosyl transferase family 1 member A7
UGT2B7: uridine diphosphate (UDP) glucuronosyl transferase family 2 member B7
UGT2B15: uridine diphosphate (UDP) glucuronosyl transferase family 2 member B15
GMDR: generalized multifactor dimensionality reduction
FDR: false discovery rate
MnBP: mono-n-butyl phthalate
DnBP: di-n-butyl phthalate
MiBP: mono-isobutyl phthalate
DiBP: diisobutyl phthalate
MBzP: mono-benzyl phthalate
BBzP: butylbenzyl phthalate
DEHP: di (2-ethylhexyl) phthalate
MEHP: mono(2-ethyl-hexyl) phthalate
MEHHP: mono(2-ethyl-5-hydroxyhexyl) phthalate
MEOHP: mono(2-ethyl-5-oxohexyl) phthalate
MECCP: mono (2-ethyl-5-carboxypentyl)phthalate
MCMHP: mono-2- carboxymethyl hexyl phthalate
LOD: limit of detection
ETS: environmental tobacco smoke
