# Supplementary material for: Association of maternal phthalates exposure and metabolic gene polymorphisms with congenital heart diseases: a multicenter case-control study
Source: BMC Pregnancy Childbirth. 2024 Feb 26;24:167. doi: 10.1186/s12884-024-06343-z (PMC10895762; doi:10.1186/s12884-024-06343-z)
Supplement: Supplementary file 1 — Supplementary Material 1 [file 12884_2024_6343_MOESM1_ESM.docx]

**Association of maternal phthalates exposure and** **metabolic gene polymorphisms with congenital heart disease: A multicenter** **case-control study**

Nana Li ^1,2^, Hong Kang ^1,2^, Zhen Liu ^1,2^, Lu Li^1,2^, Ying Deng ^1,2^, Meixian Wang ^1,2^, Yuting Li^1,2^, Wenli Xu^3^, Xiaohong Li^1,2^, Yanping Wang ^1,2^, Jun Zhu ^1,2^, Jing Tao^1,2,*^ , Ping Yu ^1,2,*^

**Appendix A:**

**Measurement of urinary concentrations of phthalate metabolites**

0.5 mL urine sample was spiked with 5 μL of mixed internal standard application solution (1 μg/mL), and then add 200 μL of ammonium acetate solution (pH 6.0) and 20 μL of β-glucuronidase, vortexed and mixed well, and enzymatically hydrolyzed at 37℃ for 16 hours. Following enzymatic hydrolysis, it was cooled to room temperature, and added 400 μL of ammonia solution (1.0%, v/v), then to be purified. After the Oasis MAX (150 mg, 6 CC) solid phase extraction column was activated with 4.0 mL methanol and 4.0 mL ultrapure water, the above supernatant was transferred to the solid phase extraction column, and then rinsed with 4.0 mL 1.0% (v/v) ammonia aqueous solution, methanol, and 30% methanol (containing 1% formic acid), and finally eluted with 5.0 mL of 1.0% formic acid methanol. The eluent was blown with nitrogen to nearly dry (50℃), reconstituted with 0.5mL of acetonitrile-aqueous solution (50:50, v/v), vortexed and mixed well, then transfered to a 1.5 mL centrifuge tube and centrifuged for 10 min (13,000 r/min, 20℃). The supernatant was taken to the sample bottle for testing.

UHPLC-MS/MS analysis was performed using an ultra-high performance liquid chromatography ACQUITY UPLC I-Class coupled to an Xevo TQ-XS triple stage quadrupole mass spectrometer (Waters, USA). The system was controlled by the MassLynx software, which also was used for the data acquisition, analysis and quantitation. The chromatographic separation of phthalate metabolites was achieved on ACQUITY UPLC BEH C18 column (2.1 mm×100 mm, 1.7 µm, Waters, USA) with a guard column (ACQUITY UPLC BEH C18 VanGuard, Waters, USA). The injection volume was 5μL, and column temperature was set at 40 °C. The mobile phase was 0.1% acetic acid in water (v/v) (A) and 0.1% acetic acid in acetonitrile (v/v) (B) at a flow rate of 0.3 mL/min. The mobile phase gradient was as follows: 30% B for 4.0 min, linear increased to 95% B from 4.0 to 9.0 min, and then maintained at 95% B from 9.0 to 10.0 min, went back to 30% B from 10.0 to 10.1 min and maintained from 10.1 to 15.0 min.

MS/MS detection was carried out with electrospray ionization (ESI) in negative ion mode using multiple-reaction monitoring (MRM) transitions and segmented monitoring for quantification of each compound. The capillary voltage was 2.5 kV and the source block temperature was 150℃. Nitrogen was used as desolvation gas, delivered at a temperature of 450℃ and a flow rate of 1000 L/h. Argon was used as collision gas at a flow rate of 0.17 mL/min. MS/MS conditions are listed in Supplementary Appendix, Table S1.

Table S1 Mass parameters and retention time of phthalates metabolites

| Chemical name | Rentention time（min） | Precursor ion (m/z) | Product ion (m/z) | Dwell time(s) | Cone(ev) | CE(V) |
| --- | --- | --- | --- | --- | --- | --- |
| MECPP | 5.62 | 307.032 | 159.022* | 0.005 | 2 | 10 |
|  |  |  | 113.052 | 0.005 | 2 | 28 |
| MECPP-13C4 | 5.62 | 311.032 | 159.013* | 0.005 | 24 | 10 |
|  |  |  | 113.047 | 0.005 | 24 | 32 |
| MEHHP | 6.13 | 293.032 | 145.038* | 0.005 | 2 | 12 |
|  |  |  | 120.918 | 0.005 | 2 | 18 |
| MEHHP-13C4 | 6.15 | 296.968 | 123.982* | 0.005 | 6 | 20 |
|  |  |  | 78.983 | 0.005 | 6 | 30 |
|  |  |  | 144.987 | 0.005 | 6 | 14 |
| MiBP | 6.23 | 221.0956 | 76.967* | 0.005 | 20 | 16 |
|  |  |  | 133.999 | 0.005 | 20 | 14 |
|  |  |  | 70.984 | 0.005 | 20 | 14 |
| MiBP-13C4 | 6.20 | 225.096 | 78.978* | 0.005 | 20 | 16 |
|  |  |  | 136.977 | 0.005 | 20 | 14 |
| MEOHP | 6.33 | 291.032 | 143.026* | 0.005 | 6 | 12 |
|  |  |  | 120.922 | 0.005 | 6 | 18 |
| MEOHP-13C4 | 6.33 | 295.096 | 143.016* | 0.005 | 22 | 12 |
|  |  |  | 123.967 | 0.005 | 22 | 16 |
|  |  |  | 78.978 | 0.005 | 22 | 30 |
| MNBP | 6.41 | 221.096 | 76.967* | 0.005 | 10 | 16 |
|  |  |  | 70.984 | 0.005 | 10 | 12 |
| MNBP-13C4 | 6.40 | 225.096 | 78.978* | 0.005 | 10 | 16 |
|  |  |  | 151.019 | 0.005 | 10 | 12 |
| MCMHP | 6.62 | 307.032 | 159.022* | 0.025 | 20 | 12 |
|  |  |  | 112.993 | 0.025 | 20 | 24 |
| MCMHP-13C4 | 6.62 | 311.032 | 159.011* | 0.005 | 20 | 14 |
|  |  |  | 113.049 | 0.005 | 20 | 26 |
| MBZP | 6.71 | 255.096 | 76.966* | 0.005 | 2 | 18 |
|  |  |  | 107.334 | 0.005 | 2 | 14 |
| MBZP-13C4 | 6.71 | 259.032 | 107.003* | 0.005 | 24 | 12 |
|  |  |  | 79.433 | 0.005 | 24 | 16 |
| MEHP | 8.79 | 276.968 | 133.981* | 0.035 | 2 | 14 |
|  |  |  | 127.058 | 0.035 | 2 | 14 |
| MEHP-13C4 | 8.79 | 281.032 | 136.984* | 0.035 | 16 | 16 |
|  |  |  | 78.918 | 0.035 | 16 | 16 |

*定量离子a Quantification ion

Table S2 The information of SNPs

| Gene | dbSNP_ID | Chr | Chr. Position* | Ref. Allele | Alt. Allele | SNP Property | Ref mRNA | AA Change | 1000 Genomes  (East Asia) |
| --- | --- | --- | --- | --- | --- | --- | --- | --- | --- |
| *UGT1A7* | rs11692021 | chr2 | 233682559 | T | C | Exonic | NM_019077 | p.W208R | C=0.1964 |
| *UGT1A7* | rs4124874 | chr2 | 233757013 | T | A,G | Intronic | NM_019077 | / | G=0.3562 |
| *UGT1A7* | rs10929302 | chr2 | 233757136 | G | A | Intronic | NM_019077 | / | A=0.1270 |
| *UGT1A7* | rs887829 | chr2 | 233759924 | C | T | Intronic | NM_019077 | / | T=0.1300 |
| *UGT1A7* | rs4148323 | chr2 | 233760498 | G | A | Exonic | NM_019077 | p.G71R | A=0.1379 |
| *UGT2B15* | rs3100 | chr4 | 68646936 | G | A | 3 Prime UTR Variant | NM_001076 | / | A=0.1895 |
| *UGT2B15* | rs4148269 | chr4 | 68647129 | T | G | Exonic | NM_001076 | p.K523T | G=0.1895 |
| *UGT2B15* | rs2045100 | chr4 | 68667984 | T | A | Intronic | NM_001076 | / | A=0.2817 |
| *UGT2B15* | rs1902023 | chr4 | 68670366 | A | C | Exonic | NM_001076 | p.Y85D | C=0.5774 |
| *UGT2B15* | rs9994887 | chr4 | 68671757 | A | G | 2KB Upstream Variant | NM_001076 | / | G=0.5744 |
| *UGT2B15* | rs13112099 | chr4 | 68672015 | T | G | 2KB Upstream Variant | NM_001076 | / | G=0.5744 |
| *UGT2B15* | rs7686914 | chr4 | 68672197 | T | A,C | 2KB Upstream Variant | NM_001076 | / | C=0.5744 |
| *UGT2B15* | rs7696472 | chr4 | 68672462 | G | A | 2KB Upstream Variant | NM_001076 | / | A=0.5744 |
| *UGT2B7* | rs4587017 | chr4 | 69081680 | T | G | Intronic | NM_001074 | / | G=0.7252 |
| *UGT2B7* | rs7662029 | chr4 | 69096194 | A | G,T | Intronic | NM_001074 | / | G=0.7242 |
| *UGT2B7* | rs12233719 | chr4 | 69096731 | G | A,C,T, | Exonic | NM_001074 | p.A71T | T=0.1319 |
| *UGT2B7* | rs10028494 | chr4 | 69105219 | A | C,T | Intronic | NM_001074 | / | C=0.2103 |
| *CYP2C19* | rs12248560 | chr10 | 94761900 | C | A,T | 2KB Upstream Variant | NM_000769 | / | T=0.0149 |
| *CYP2C19* | rs4244285 | chr10 | 94781859 | G | A,C | Exonic | NM_000769 | p.P227P | A=0.3222 |
| *CYP2C9* | rs1057910 | chr10 | 94981296 | A | C,G | Exonic | NM_000771 | p.I359L | C=0.0337 |

*The reference genome information is Hg38.

Table S3. Logistic regression analyses of the association between phthalate metabolites in maternal urinary samples and the risk of CHDs in the Han Chinese maternal population

| Elements | Concentration levels ^a^ | Controls |  | CHDs Cases | cOR (95% CI) | aOR (95% CI)^b^ |
| --- | --- | --- | --- | --- | --- | --- |
|  |  | No. (%) |  | No. (%) |  |  |
| MiBP | First-tertile | 86(33.20) |  | 64(33.86) | Reference | Reference |
|  | Second-tertile | 85(32.82) |  | 66(34.92) | 1.04(0.66-1.65) | 0.95(0.58-1.56) |
|  | Third-tertile | 88(33.98) |  | 59(31.22) | 0.90(0.57-1.43) | 0.80(0.49-1.32) |
| MnBP | First-tertile | 89(34.36) |  | 60(31.75) | Reference | Reference |
|  | Second-tertile | 89(34.36) |  | 59(31.22) | 0.98(0.62-1.56) | 1.08(0.65-1.80) |
|  | Third-tertile | 81(31.27) |  | 70(37.04) | 1.28(0.81-2.03) | 1.35(0.82-2.23) |
| MBzP | First-tertile | 84(32.43) |  | 65(34.39) | Reference | Reference |
|  | Second-tertile | 91(35.14) |  | 57(30.16) | 0.81(0.51-1.29) | 0.76(0.46-1.25) |
|  | Third-tertile | 84(32.43) |  | 67(35.45) | 1.03(0.65-1.63) | 1.01(0.61-1.66) |
| MEHP | First-tertile | 76(29.34) |  | 73(38.62) | Reference | Reference |
|  | Second-tertile | 94(36.29) |  | 55(29.10) | 0.61(0.38-1.01) | 0.51(0.31-1.02) |
|  | Third-tertile | 89(34.36) |  | 61(32.28) | 0.71(0.45-1.13) | 0.62(0.38-1.03) |
| MEHHP | First-tertile | 90(34.75) |  | 58(30.69) | Reference | Reference |
|  | Second-tertile | 90(34.75) |  | 54(28.57) | 0.93(0.58-1.49) | 0.78(0.47-1.29) |
|  | Third-tertile | 79(30.50) |  | 77(40.74) | 1.51(0.96-2.38) | 1.33(0.81-2.18) |
| MEOHP | First-tertile | 91(35.14) |  | 57(30.16) | Reference | Reference |
|  | Second-tertile | 88(33.98) |  | 63(33.33) | 1.14(0.72-1.82) | 0.93(0.56-1.53) |
|  | Third-tertile | 80(30.89) |  | 69(36.51) | 1.38(0.87-2.19) | 1.20(0.73-1.99) |
| MECPP | First-tertile | 92(35.52) |  | 55(29.10) | Reference | Reference |
|  | Second-tertile | 87(33.59) |  | 65(34.39) | 1.25(0.79-1.99) | 0.97(0.58-1.59) |
|  | Third-tertile | 80(30.89) |  | 69(36.51) | 1.44(0.91-2.29) | 1.15(0.69-1.91) |
| MCMHP | First-tertile | 84(32.43) |  | 64(33.86) | Reference | Reference |
|  | Second-tertile | 98(37.84) |  | 51(26.98) | 0.68(0.43-1.09) | 0.54(0.32-1.07) |
|  | Third-tertile | 77(29.73) |  | 74(39.15) | 1.26(0.80-1.99) | 1.11(0.68-1.83) |

a. Data were divided by overall maternal urine tertiles log10-transformed phthalate metabolites concentrations.

b. aOR, adjusted odds ratio. Logistic regression was used to calculate odds ratios and 95% CIs; all models were adjusted for maternal age (continuous), maternal ethnicity, maternal education level, parental smoking or ETS exposure, maternal alcohol consumption, gravidity, pre-pregnancy BMI (continuous), folic acid supplements.

Table S4 Hardy-Weinberg equilibrium evaluation of SNPs

| Gene | dbSNP_ID | Group | Genotype count（frequency %） | | | HWE *P* |
| --- | --- | --- | --- | --- | --- | --- |
| *UGT1A7* |  |  | T/T | T/C | C/C |  |
|  | rs11692021 | Control | 154(57.46) | 98(36.57) | 16(5.97) | 1 |
|  | rs11692021 | Case | 159(64.9) | 75(30.61) | 11(4.49) |  |
| *UGT1A7* |  |  | T/T | T/G | G/G |  |
|  | rs4124874 | Control | 142(52.99) | 108(40.3) | 18(6.72) | 0.757 |
|  | rs4124874 | Case | 96(39.18) | 112(45.71) | 37(15.1) |  |
| *UGT1A7* |  |  | G/G | G/A | A/A |  |
|  | rs10929302 | Control | 215(80.22) | 52(19.4) | 1(0.37) | 0.4924 |
|  | rs10929302 | Case | 194(79.18) | 42(17.14) | 9(3.67) |  |
| *UGT1A7* |  |  | C/C | C/T | T/T |  |
|  | rs887829 | Control | 214(79.85) | 53(19.78) | 1(0.37) | 0.3301 |
|  | rs887829 | Case | 194(79.18) | 42(17.14) | 9(3.67) |  |
| *UGT1A7* |  |  | G/G | G/A | A/A |  |
|  | rs4148323 | Control | 174(64.93) | 81(30.22) | 13(4.85) | 0.3448 |
|  | rs4148323 | Case | 181(73.88) | 59(24.08) | 5(2.04) |  |
| *UGT2B15* |  |  | G/G | G/A | A/A |  |
|  | rs3100 | Control | 193(72.01) | 69(25.75) | 6(2.24) | 1 |
|  | rs3100 | Case | 160(65.31) | 78(31.84) | 7(2.86) |  |
| *UGT2B15* |  |  | T/T | T/G | G/G |  |
|  | rs4148269 | Control | 192(71.64) | 70(26.12) | 6(2.24) | 1 |
|  | rs4148269 | Case | 160(65.31) | 78(31.84) | 7(2.86) |  |
| *UGT2B15* |  |  | T/T | T/A | A/A |  |
|  | rs2045100 | Control | 125(46.64) | 119(44.4) | 24(8.96) | 0.6692 |
|  | rs2045100 | Case | 119(48.57) | 104(42.45) | 22(8.98) |  |
| *UGT2B15* |  |  | C/C | C/A | A/A |  |
|  | rs1902023 | Control | 84(31.34) | 124(46.27) | 60(22.39) | 0.2696 |
|  | rs1902023 | Case | 85(34.69) | 115(46.94) | 45(18.37) |  |
| *UGT2B15* |  |  | G/G | G/A | A/A |  |
|  | rs9994887 | Control | 85(31.72) | 123(45.9) | 60(22.39) | 0.2202 |
|  | rs9994887 | Case | 83(33.88) | 116(47.35) | 46(18.78) |  |
| *UGT2B15* |  |  | G/G | G/T | T/T |  |
|  | rs13112099 | Control | 85(31.72) | 123(45.9) | 60(22.39) | 0.2202 |
|  | rs13112099 | Case | 83(33.88) | 116(47.35) | 46(18.78) |  |
| *UGT2B15* |  |  | T/T | C/T | C/C |  |
|  | rs7686914 | Control | 60(22.39) | 123(45.9) | 85(31.72) | 0.2202 |
|  | rs7686914 | Case | 46(18.78) | 116(47.35) | 83(33.88) |  |
| *UGT2B15* |  |  | G/G | A/G | A/A |  |
|  | rs7696472 | Control | 60(22.39) | 123(45.9) | 85(31.72) | 0.2202 |
|  | rs7696472 | Case | 46(18.78) | 115(46.94) | 84(34.29) |  |
| *UGT2B7* |  |  | T/T | G/T | G/G |  |
|  | rs4587017 | Control | 19(7.09) | 102(38.06) | 147(54.85) | 0.8741 |
|  | rs4587017 | Case | 22(8.98) | 93(37.96) | 130(53.06) |  |
| *UGT2B7* |  |  | A/A | G/A | G/G |  |
|  | rs7662029 | Control | 19(7.09) | 106(39.55) | 143(53.36) | 1 |
|  | rs7662029 | Case | 22(8.98) | 94(38.37) | 129(52.65) |  |
| *UGT2B7* |  |  | T/T | G/T | G/G |  |
|  | rs12233719 | Control | 9(3.36) | 75(27.99) | 184(68.66) | 0.6711 |
|  | rs12233719 | Case | 177(72.24) | 61(24.9) | 7(2.86) |  |
| *UGT2B7* |  |  | A/A | A/C | C/C |  |
|  | rs10028494 | Control | 166(61.94) | 94(35.07) | 8(2.99) | 0.2641 |
|  | rs10028494 | Case | 152(62.04) | 84(34.29) | 9(3.67) |  |
| *CYP2C19* |  |  | C/C | C/T | T/T |  |
|  | rs12248560 | Control | 262(97.76) | 6(2.24) | 0(0) | 1 |
|  | rs12248560 | Case | 239(97.55) | 6(2.45) | 0(0) |  |
| *CYP2C19* |  |  | G/G | G/A | A/A |  |
|  | rs4244285 | Control | 118(44.03) | 121(45.15) | 29(10.82) | 0.891 |
|  | rs4244285 | Case | 108(44.08) | 105(42.86) | 32(13.06) |  |
| *CYP2C9* |  |  | A/A | A/C | C/C |  |
|  | rs1057910 | Control | 256(95.52) | 11(4.1) | 1(0.37) | 0.1384 |
|  | rs1057910 | Case | 225(91.84) | 20(8.16) | 0(0) |  |
